# Modelling the effect of the inhibitors on asphaltene precipitation using Flory–Huggins theory

**DOI:** 10.1038/s41598-022-23596-w

**Published:** 2022-11-08

**Authors:** Farzaneh Eskini, Amirhossein Saeedi Dehaghani, Mohammad Mahdi Shadman

**Affiliations:** 1 Department of Petroleum Engineering, Faculty of Chemical Engineering, Tarbiat Modares University, Tehran, Iran; 2 Nuclear Fuel Cycle Research School, Nuclear Science and Technology Research Institute, Tehran, Iran

**Keywords:** Chemical engineering, Polymer chemistry

## Abstract

Due to the technical, environmental and economic problems caused by asphaltene precipitation, such as oil production reduction, well shut-ins and the necessity of EOR usage, the prediction of asphaltene precipitation seems to be vital. Considering the larger size of asphaltene molecules compared to the other hydrocarbon, it is reasonable to predict the precipitation using the Flory–Huggins theory. In this study, Flory–Huggins solution theory has been modified regarding the solvent molar volume. The modified model was used to predict the asphaltene precipitation of four oil samples in the absence and presence of the inhibitors. Then, the modeling data given by the Flory–Huggins theory was validated with the experimental data obtained by ASTM D-6560 standard method. The mean error at this modeling was 2–13%, which seems acceptable. The proposed model for the cases where an inhibitor is not involved has higher accuracy. The modified Flory–Huggins theory confirmed that the addition of inhibitors at all concentrations postpones the onset point. The average error of the modified model was found to be 4.5–9.8%, which is in a good range. Also, the model accuracy is less for situations where the asphaltene content of the crude oil is higher. Based on this study, the modification of Flory–Huggins theory, regarding the solvent molar volume leads to a lower error.

## Introduction

Asphaltene is described as a fraction of crude oil which is soluble in light aromatics but insoluble in paraffins^1,2^. Deposition of asphaltene always causes technical and economic problems in oil wells. It is important to mention that there is a difference between precipitation and deposition of asphaltene. Precipitation is when a semi-solid phase is formed. The deposition is when asphaltene forms on the solid surface. Precipitation will not necessarily lead to deposition, but it will affect it. Asphaltene precipitation is a function of changes in pressure, temperature, and the composition of the crude oil, but deposition is a process that occurs after precipitation, depending on the amount of attraction and absorption between aggregates^3–6^.

Using inhibitors to postpone the precipitation process is an economic and accepted way of facing this problem. When inhibitors are added to the cured oil, the solubility of the system is changed. Although the asphaltene molecules are much bigger than the other molecules in the crude oil, It is possible to use polymeric solution theories to model it^7,8^. Flory–Huggins theory, as a theory that considers and uses the solubility parameters of the mixtures components, is a good choice to model asphaltene precipitation in the presence of inhibitors^9^. Using other models such as PC-SAFT and CPA for such a system will miss this point^10^.

For the first time, Hirschberg attempted to implement the Flory–Huggins theory to predict the precipitation behavior of asphaltene. In 1948, Hirschberg et al. proposed a thermodynamic model to describe the behavior of asphaltene in crude oil under the influence of temperature change, pressure, or component composition^11,12^. In their model, they used Flory–Huggins theory along with the Soave equation. They assumed a vapor–liquid equilibrium, but the Benedict–Webb–Rubin equation was used to obtain molar volume. In their model, oil was assumed to be comprised of two components; the asphaltene free fraction and asphalt, which is a combination of asphaltene and resin. Another assumption was that the volume ratio of asphaltene is equal to the maximum volume ratio of the asphaltene molecules at the asphaltene precipitation point φ_a_, i.e.^11,13^. In 2006, Mofidi and Edalat calculated the threshold of asphaltene precipitation in the presence of different solvents using the polymer solubility model. By applying Flory–Huggins thermodynamic theory to derive the Gibbs free energy relationship, the asphaltene precipitants were obtained through solving the equations given by minimizing Gibbs free energy. Because the molecular weight of asphaltene varies in different solvents, they adjusted the molecular weight of asphaltene using experimental data^14^. In 2006, Pazuki and Nikokar modified the Flory–Huggins model to predict the phase behavior of the asphaltene precipitation by adding solvents such as normal pentane, normal hexane, and normal heptane to an oil sample. They assumed a vapor-liquid-liquid equilibrium and used the concept of mass balance to modify the model. The Soave-Redlich-Kwong equation of state was used to estimate component properties in lighter fractions. The equilibrium calculations and the properties of the heavier parts in the crude oil system were measured by experimental correlations^15^.

Daryasafar et al.^10^ performed a comparative study to compare the prediction accuracy of the cubic-plus-association (CPA), perturbed-chain statistical associating fluid theory (PC-SAFT), solid model, Flory–Huggins, and the modified Flory Huggins models. Based on their study, which was conducted on twelve different oil samples, the PC-SAFT and CPA models had better accuracy in predicting the asphaltene precipitation^10^.

Here in this study, first volume shift parameters for modifying the molar volume of the solvent, which includes all the crude oil except for the asphaltene, were used to adjust and predict the asphaltene precipitation amount in the absence of the inhibitors. Then assuming that adding inhibitors to the system will change the solubility parameter of the solvent, and by applying Flory–Huggins theory, the model has been proposed. As mentioned before, using Flory–Huggins theory in such a system of crude oil and inhibitors and considering the importance and role of a solubility parameter change is a point that has not been seen in the literature before.

## Modeling and experimental approaches

As mentioned before, the Flory–Huggins method, as a common model to explain the thermodynamics of polymer dissolution, was modified to predict the asphaltene precipitation in the presence and absence of the inhibitors. To investigate the validity of the implemented method, the experimental data of filtration and viscometric tests were exploited.

### Fluid properties

#### Crude oil samples

The oil samples used in this study originated from several oil fields in the South of Iran. The API density and locations of these oil fields are listed in Table 1.

**Table 1:** location and API density of crude oil samples.

Location/ formation	API	Crude oil sample
Azadegan, Sarvak formation	25.57	Sample A
Ahvaz, Bangistan, Ilam formation	25.84	Sample B
Ab-Teymour, Sarvak formation	26.42	Sample C
Cheshme khosh	25.20	Sample D

The asphaltene content of the oil sample was determined by using ASTM D-6560 standard method. Besides, the saturate, aromatic, and resin fractions of the oil sample were determined by conducting the Saturate-Aromatic-Resin-Asphaltene (SARA) test. The composition, resin, and asphaltene contents of the oil samples are shown in Table 2.

**Table 2:** The composition, resin, and asphaltene content of the oil samples.

Component/Sample	Sample A	Sample B	Sample C	Sample D
C_2_	0	0.10	0.10	0.09
C_3_	0.25	0.14	0.27	0.12
i-C_4_	0	0.26	0.26	0.14
n-C_4_	1.57	1.69	1.50	1.06
i-C_5_	0	1.61	1.67	1.93
n-C_5_	4.91	1.98	1.96	2.15
C_6_	6.77	11.70	10.60	10.70
C_7_	8.04	7.64	5.32	7.60
C_8_	7.56	6.82	5.56	6.83
C_9_	7.34	10.20	6.48	9.58
C_10_	5.44	5.63	5.32	6.29
C_11_	4.30	6.11	5.56	5.91
ps-1	7.27	6.61	6.48	7.58
ps-2	2.58	2.35	2.30	2.69
ps-3	1.19	1.08	1.06	1.24
ps-4	0.13	0.11	0.11	0.13
ps-5	30.50	27.70	27.2	31.80
Asphaltene	2.32	1.42	1.71	0.81
resin	9.83	6.85	9.45	3.36

#### Inhibitor properties

In this study, economical industrial compounds, which have inhibitory properties and are compatible with crude oil, have been used. The individual effect of the 15 inhibitors was investigated. The inhibitors and corresponding concentrations used as inhibitor mixture is listed in Table 3.

**Table 3:** The inhibitors and corresponding concentrations used as inhibitor mixture.

Concentration	Sample A	Sample B	Sample C	Sample D
0	Without inhibitor	Without inhibitor	Without inhibitor	Without inhibitor
500	B3	B14	B13	B4
5000	B4	B13	B14	B3
10,000	B13	B4	B3	B14
20,000	B14	B3	B4	B13

According to the analysis we have done before by applying Latin Square Design method if we assume the concentration of the inhibitor as a factor A, the type of the inhibitors as factor B, and the crude oil sample as factor C, there are no interactions between A/C or B/C. So, the type of crude oil has no effect on the efficiency of the inhibitor^16,17^. So just the inhibitors at different concentrations in different oil samples have been examined.

After conducting the turbidity test according to the design experiment table, the most effective inhibitors, including inhibitors of Coconut Diethanolamide, Cocamide monoethanolamine, branched dodecyl benzene sulfonic acid, and linear dodecyl benzene sulfonic acid, was selected (B3, B4, B13, B14) at the concentrations of 500, 5000, 10,000, 20,000 ppm. Because the interactions of AC and BC are not significant and a similar trend was observed for an inhibitor in different oils, the method can be used. Viscometric and separation with filter paper are designed according to Table 3.

### Experimental methods

The Asphaltene content of an oil sample can not be completely precipitated by adding solvents. The Asphaltene data of this study has been resulted of ASTM D-6560 method. Also SARA experiment has been done to characterize saturate, aromatic, resin and Asphaltene contents of the oil sample. The inhibitors at different concentrations from 500 to 20,000 ppm get prepared by using air displacement pipette. Preparation of these solutions was at the situation of 50 °C and 1 h in shaker.

Next step in the procedure was determining of the onset point using viscometric test.The viscosities of the mixtures of oil and the solvent were evaluated by using the Stabinger viscometer (Anton Paar, Austria). By measuring the oil mixtures containing various solvent concentrations, the onset point for the asphaltene precipitation was determined. Before conducting the viscometric tests, the oil + solvent mixture was kept in a shaker for 1 h, leading to stabilizing the mixture. All the viscometric tests were conducted at 20 °C. It should be mentioned that the accuracy of the Stabinger viscometer lies in the range of 0.2–20,000 m.Pa.s for the dynamic viscosities. The procedure used for viscometric tests is similar to the process that Fazeli et al.^18^ conducted to study the viscoelastic behavior of the asphaltenic crude oil regarding the angular frequency and N-heptane concentration^18^.

The filtration tests were conducted to investigate the precipitant asphaltenes in compliance with the ASTM D2007-80 standard. First, based on the viscometric tests, the asphaltene onset point was determined. Then different amounts of normal heptane were added to 5 g of dead oil and inhibitors. The mixtures of oil + solvent were stirred on a magnetic stirrer to entirely mix the oil and solvent. Afterward, the mixtures were kept in the dark place to let the asphaltene clusters grow. Finally, the asphaltene precipitants were filtered using the Whatman filter papers (#42 with the pore size of 47 $\mathrm{\mu m}$) via a vacuum pump. To improve the accuracy of the filtration, the asphaltene cake was washed with 5 ml of normal heptane. The asphaltene mass was determined after drying the filter at 75 °C for 1 h. Again the filter papers were weighted and the results were reported as the asphaltene weight percent.

### Modeling approach

The modeling process used in this study can be divided into two parts. One is the modeling for the condition in which the experiments were performed in the absence of an inhibitor. This part is the base of the study, and it is used to compare the efficiency of the inhibitor. The other is the process in which the inhibitor was used in different concentrations and solvent ratios. The solvent ratio stands for the mixing ratio of the solvent and the crude oil. In the end, the graphs of these two studies will be compared to determine the inhibitor's efficiency.

#### Modeling in the absence of inhibitors

**Figure 1:**
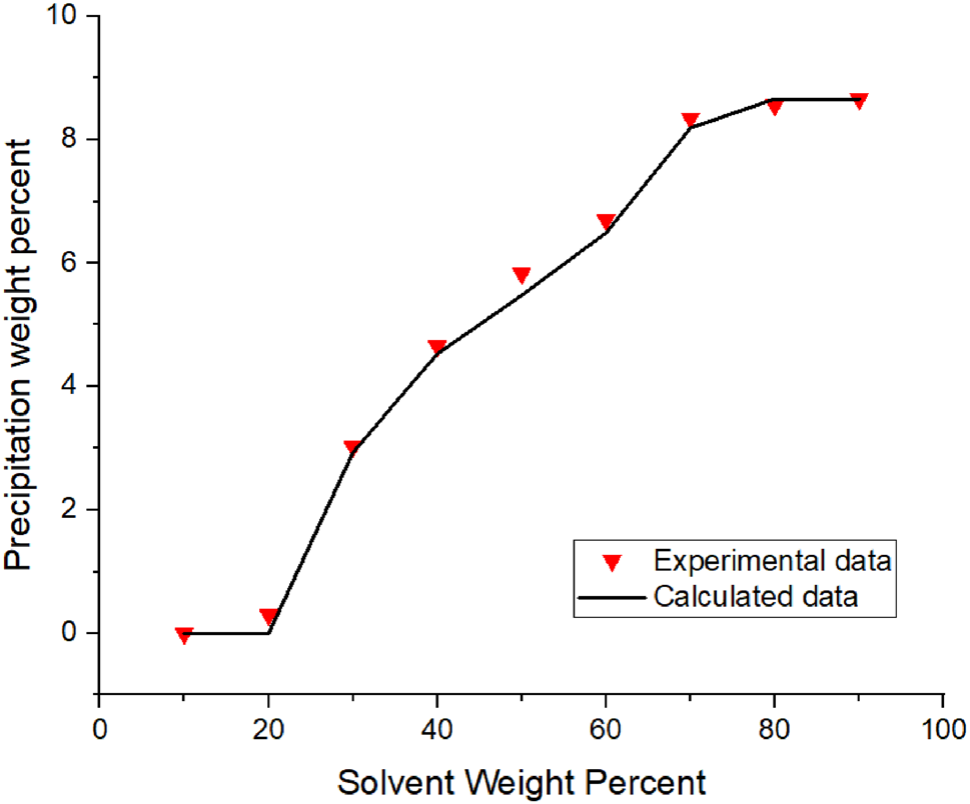
Asphaltene precipitation from the oil sample A versus solvent percent: obtained from experimental and modeling approaches.

The algorithm, which is shown in Fig. 1A (Appendix), summarizes the modeling approach implemented to predict the asphaltene precipitation in the absence of the inhibitors. The details of how and why equations are used are explained in the schemes represented in the Appendix.

First of all, after taking the initial properties of the crude oil samples, such as: temperature, pressure, critical pressure and critical temperatures, molar volume of the samples in the absence of inhibitors was calculated by Peng Robinson equation.

1 $$\mathrm{P}=\frac{\mathrm{RT}}{\mathrm{v}-\mathrm{b}}-\frac{\mathrm{a}\left(\mathrm{T}.\mathrm{ w}\right)}{\mathrm{v}\left(\mathrm{v}+\mathrm{b}\right)+\mathrm{b}\left(\mathrm{v}-\mathrm{b}\right)}$$

Then the solubility of asphaltene was calculated from the correlation used in Yazdizadeh's work^19^.

2 $${\updelta }_{\mathrm{a}}=\left\{1-1.07\times {10}^{-3}\left(\mathrm{T}-273.15)\}\right.\right.$$

For calculating the solubility parameter of the solvent phase, the following equation has been derived from Peng Robinson EOS:

3 $${\updelta }_{\mathrm{m}}=\sqrt{\frac{{\mathrm{U}}^{\mathrm{vap}}\left(\mathrm{T},\mathrm{P}=0\right)-{\mathrm{U}}^{\mathrm{liq}}\left(\mathrm{T}-\mathrm{P}\right)}{{\mathrm{v}}_{\mathrm{m}}} }=\sqrt{[\mathrm{T}\frac{{\mathrm{da}}_{\mathrm{m}}}{\mathrm{dT}}-{\mathrm{a}}_{\mathrm{m}}]\left(\frac{\sqrt{\mathrm{a}}}{46\mathrm{m}}\right)\mathrm{ln}\left(\frac{{\mathrm{v}}_{\mathrm{m}}+(1+\sqrt{2}){\mathrm{b}}_{\mathrm{m}}}{{\mathrm{v}}_{\mathrm{m}}+(1-\sqrt{2}){\mathrm{b}}_{\mathrm{m}}}\right)}$$

Asphaltene partial volume is calculated by the equation from Mofidi-Edalat's work^14^.

4 $$\mathrm{ln}{\mathrm{\varphi }}_{\mathrm{a}}^{\mathrm{s}}+\left(1-{\mathrm{\varphi }}_{\mathrm{a}}^{\mathrm{s}}\right)\left(1-\frac{{\mathrm{V}}_{\mathrm{a}}}{{\mathrm{V}}_{\mathrm{s}}}\right)+{\left(1-{\mathrm{\varphi }}_{\mathrm{a}}^{\mathrm{s}}\right)}^{2}\frac{{\mathrm{V}}_{\mathrm{a}}}{\mathrm{RT}}{\left({\updelta }_{\mathrm{a}}-{\updelta }_{\mathrm{s}}\right)}^{2}=0$$

Finally Asphaltene's weight fraction was calculated from the equation used in Mofidi-Edaat work to be compared by the experimental value. ^14^.

5 $$\mathrm{w}=\frac{\left(1-{\mathrm{\varphi }}_{\mathrm{a}}^{\mathrm{s}}\right)\left(\frac{{\mathrm{Mw}}^{\mathrm{s}}}{{\mathrm{V}}_{\mathrm{s}}}\right)}{\left(1-{\mathrm{\varphi }}_{\mathrm{a}}^{\mathrm{s}}\right)\frac{{\mathrm{Mw}}^{\mathrm{a}}}{{\mathrm{V}}_{\mathrm{a}}}+{\mathrm{\varphi }}_{\mathrm{a}}^{\mathrm{s}}\frac{{\mathrm{Mw}}^{\mathrm{s}}}{{\mathrm{V}}_{\mathrm{s}}}}$$

**Figure 2:**
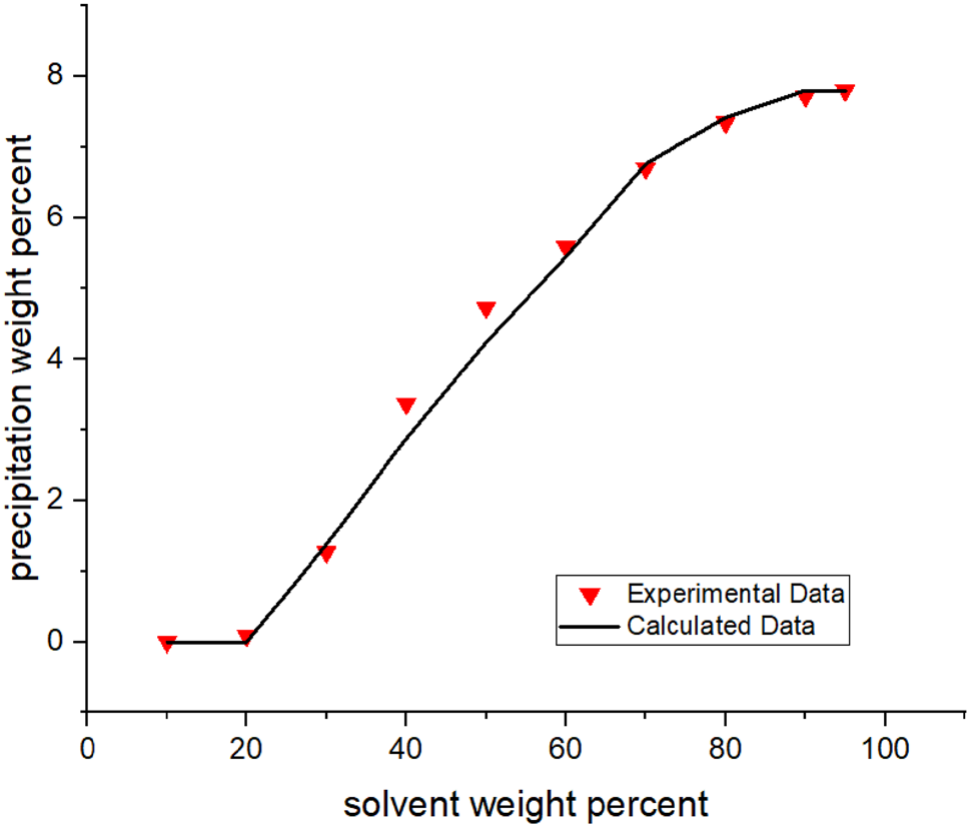
Asphaltene precipitation from the oil sample B versus solvent percent: obtained from experimental and modeling approaches.

In this procedure, Flory–Huggin's theory is used to calculate φ_a_. In the meantime, some hypotheses have been used to use the theory. Two phases are considered: one is the precipitate phase (p), and the other is the oil or solvent phase (s). In this study, the precipitant phase is considered as pure asphaltene, as in previous research conducted by Hirschberg et al. ^11^ and Pazuki et al.^15^. With this assumption, the changing rate of the chemical potential of asphaltene **(**Δμ_a_**)** in the asphaltene phase is zero. The interaction parameter is another factor involved in the application of this theory. First By using this parameter in Flory–Huggin's theory, the response of the calculation did not converge. Therefore, the value of this parameter was set to zero. (Fig. 1A in the Appendix) Secondly by applying this assumption, the equilibrium condition for calculating the amount of asphaltene precipitation is obtained by Eq. (4). The algorithm error was relatively high if only the volume shift was set. Hence, the process was modified by adjusting the interaction parameter.(Fig. 2A in Appendix) In this regard, Eq. 6, which was used by Yazdizadeh et al.^19^, was implemented to determine the interaction parameter**.**

6 $$\left\{\begin{array}{c}{\mathbf{l}}_{\mathbf{a}\mathbf{s}}={\mathbf{a}}_{0}+{\mathbf{a}}_{1}F+{\mathbf{a}}_{2}{\mathbf{F}}^{2}+{\mathbf{a}}_{3}{\mathbf{F}}^{3}\\ F=\frac{(1+\mathbf{S}\mathbf{R}){\mathbf{M}\mathbf{w}}_{\mathbf{a}}\left(\frac{{\mathbf{v}}_{\mathbf{a}}^{\mathbf{o}\mathbf{i}\mathbf{l}}}{{\mathbf{v}}_{\mathbf{s}}^{\mathbf{o}\mathbf{i}\mathbf{l}}}\right)}{\mathbf{S}\mathbf{R}{\mathbf{M}\mathbf{w}}_{\mathbf{s}}\left(1+\frac{{\mathbf{v}}_{\mathbf{a}}^{\mathbf{o}\mathbf{i}\mathbf{l}}}{{\mathbf{v}}_{\mathbf{s}}^{\mathbf{o}\mathbf{i}\mathbf{l}}}\right)}\end{array}\right.$$

7 $$\mathbf{ln}{\mathbf{\varphi }}_{\mathbf{a}}^{\mathbf{s}}+\left(1-{\mathbf{\varphi }}_{\mathbf{a}}^{\mathbf{s}}\right)\left(1-\frac{{\mathbf{V}}_{\mathbf{a}}}{{\mathbf{V}}_{\mathbf{s}}}\right)+{\left(1-{\mathbf{\varphi }}_{\mathbf{a}}^{\mathbf{s}}\right)}^{2}\frac{{\mathbf{V}}_{\mathbf{a}}}{\mathbf{R}\mathbf{T}}{[\left({\updelta }_{\mathbf{a}}-{\updelta }_{\mathbf{s}}\right)}^{2}+{\mathbf{l}}_{\mathbf{a}\mathbf{s}}]=0$$

**Figure 3:**
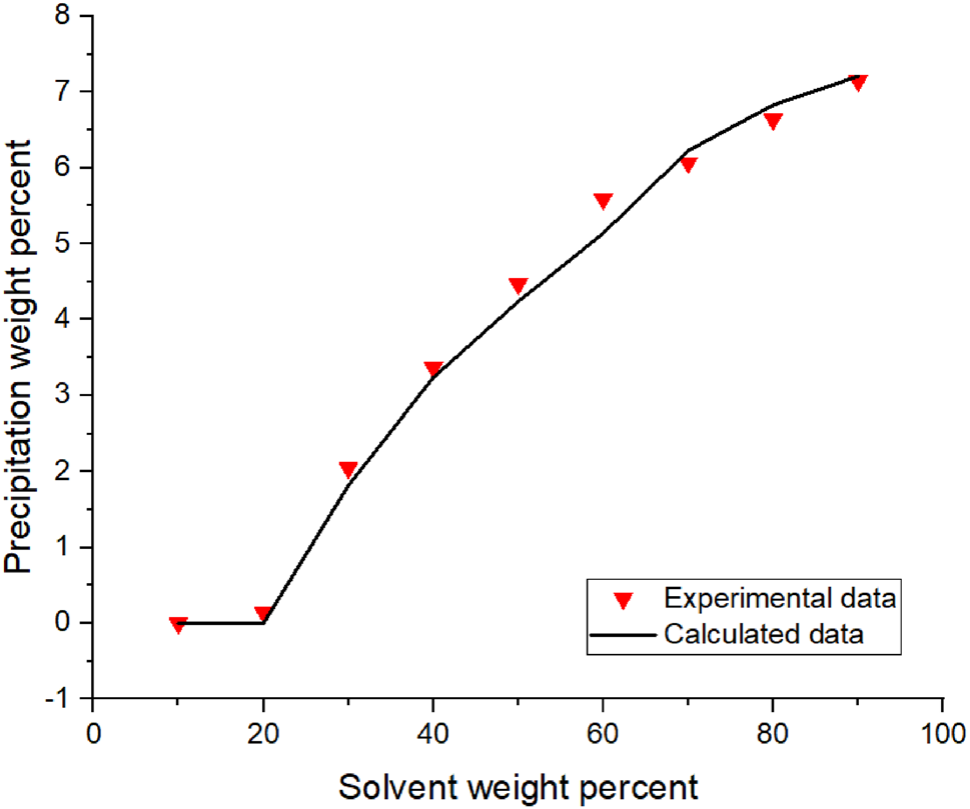
Asphaltene precipitation from the oil sample C versus solvent percent: obtained from experimental and modeling approaches.

The procedure, shown in Fig. 3A in Appendix, was used to calculate the adjusted volume shift**.** The final volume shifts for different oil samples are reported in Table 4.

**Table 4:** The Volume shifts for different oil samples.

Solvent ratio	Sample A	Sample B	Sample C	Sample D
10	17.74	37.76	30.6	92.74
20	17.41	33.33	27.23	74.56
30	17.28	30.02	24.73	60.47
40	17.17	27.45	22.8	49.23
50	16.99	25.39	21.25	40.05
60	16.86	23.71	20	32.42
70	16.75	27.32	18.96	25.97
80	16.66	21.13	18.08	20.45
90	16.59	20.12	17.33	15.67

#### Modeling in the presence of inhibitors

This section uses the Peng–Robinson equation of state, which was adjusted in the previous section. To consider the degree of inhibition effect, the solubility parameter was adjusted; the inhibitory effect on asphaltene was modeled as a change in the solubility parameter of the solvent. Because considering the inhibitor as a single component, due to its small effect on the molar volume of the solvent, it does not explicitly transmit the inhibitor and has little effect. The change in the solubility parameter was considered as the following function:

8 $$\left\{\begin{array}{c}{\updelta }_{\mathbf{s},\mathbf{c}\mathbf{o}\mathbf{r}\mathbf{r}\mathbf{e}\mathbf{c}\mathbf{t}\mathbf{e}\mathbf{d}}={\updelta }_{\mathbf{s}}-\Delta s\\ \Delta s=a. ppm. {(\frac{\mathbf{v}}{1000})}_{\mathbf{s}}^{\mathbf{b}}\end{array}\right.$$

The modeling scheme of the asphaltene precipitation prediction in the presence of the inhibitors is shown in Fig. 3A (Appendix). In this approach first, as it was done in the previous section for modelling in the abcense of inhibitors, molar volume of the asphaltene was calculated from equation number 1 and the Asphaltene solubility parameter from equation number 2. Here, using proposed equation number 8, a and b parameters for calculating solubility parameter are guessed. Then new molar ratio for the solvent including inhibitors, new molar volume of solvent and new molecular weight of the solvent are calculated. Finding new solubility parameter for the solution by the equation number 3, then modifying it, applying equation number 8 will lead to a new weight fraction for Asphaltene. Comparing this fraction with the experimental one helped us to adjust a and b parameters.

## Results and discussions

Implementing the algorithms mentioned in Figs. 1-3A, the proposed model was used, and the results were obtained. The results are given in Tables 5, 6, 7 and 8 and Figs. 1, 2, 3, 4, 5, 6, 7 and 8.

**Table 5:** Comparison of experimental and predicted asphaltene precipitation—Crude oil sample A (NRMSE = 9.8%).

Solvent ratio	Exp 0	Predicted 0	Exp 5000	Predicted 5000	Exp 10,000	Predicted 10,000	Exp 20,000	Predicted 20,000
10	0	0	0	0	0	0	0	0
20	0.3	0	0.35	0	0.2	0	0	0
30	3.03	2.94	3.35	1.46	2.97	0.89	0.4	0.46
40	4.65	4.53	4.82	4.35	5.55	4.21	3.9	3.95
50	5.83	5.48	6.96	5.44	7.1	5.43	7.52	5.40
60	6.7	6.49	7.82	6.46	7.99	6.46	8.57	6.43
70	8.35	8.19	8.97	8.15	9.32	8.11	9.47	8.02
80	8.56	8.66	8.97	8.62	9.47	8.57	9.58	8.49
90	8.66	8.66	9.04	8.62	9.52	8.57	9.60	8.48
NRMSE	2%		11%		13%		12%

**Table 6:** Comparison of experimental and predicted asphaltene precipitation—Crude oil sample B (NRMSE = 5.2%).

Solvent ratio	Exp 0	predicted 0	Exp 5000	predicted 5000	Exp 10,000	predicted 10,000	Exp 20,000	predicted 20,000
10	0	0	0	0	0	0	0	0
20	0.09	0	0	0	0	0	0	0
30	1.27	1.38	0.9	0.76	0.9	0.47	0.15	0.26
40	3.37	2.88	2.56	2.58	2.85	2.32	1.94	1.97
50	4.73	4.24	4.86	4.16	4.70	4.05	4.99	4.9
60	5.59	5.45	6.21	5.43	6.16	5.38	6.28	5.3
70	6.7	6.76	6.95	6.75	6.82	6.7	7.16	6.63
80	7.35	7.42	7.92	7.38	7.61	7.35	8.04	7.28
90	7.72	7.8	8.33	7.76	8.11	7.72	8.67	7.64
95	7.8	7.8	8.47	7.76	8.20	7.72	8.74	7.64
NRMSE	3%		6%		5%		7%

**Table 7:** Comparison of experimental and predicted asphaltene precipitation—Crude oil sample C (NRMSE = 4.5%).

Solvent ratio	Exp 0	predicted 0	Exp 5000	predicted 5000	Exp 10,000	predicted 10,000	Exp 20,000	Predicted 20,000
10	0	0	0	0	0	0	0	0
20	0.15	0	0	0	0	0	0	0
30	2.05	1.82	0.82	0.95	0.46	0.59	0.272	0.29
40	3.37	3.24	2.67	2.74	2.31	2.37	1.81	1.86
50	4.47	4.24	4.06	4.08	3.97	3.92	3.78	3.67
60	5.59	5.14	5.37	5.08	5.33	5.02	5.28	4.92
70	6.07	6.23	6.70	6.2	6.85	6.17	7.21	6.1
80	6.64	6.83	7.31	6.8	7.46	6.76	7.76	6.7
95	7.15	7.21	7.48	7.17	7.63	7.14	7.82	7.06
NRMSE	3%		4%		5%		7%

**Table 8:** Comparison of experimental and predicted asphaltene precipitation—Crude oil sample D (NRMSE = 6.7%).

Solvent ratio	Exp 0	predicted 0	Exp 5000	predicted 5000	Exp10000	predicted 10,000	Exp 20,000	predicted 20,000
10	0	0	0	0	0	0	0	0
20	0	0	0	0	0	0	0	0
30	0	0	0	0	0	0	0	0
40	0.354	0.67	0.22	0.14	0.12	0.06	0	0
50	2.279	1.46	1.83	0.95	0.96	0.67	0.381	0.4
60	3.429	2.71	3.61	2.42	3.02	2.19	1.838	1.83
70	4.447	4.42	4.53	4.3	4.67	4.15	4.85	3.88
80	5.022	5.52	5.11	5.49	5.44	5.47	5.64	5.43
90	5.641	5.71	5.68	5.68	5.85	5.65	5.98	5.59
95	5.708	5.71	5.68	5.68	5.99	5.65	6.19	5.59
NRMSE	7%		8%		6%		6%

**Figure 4:**
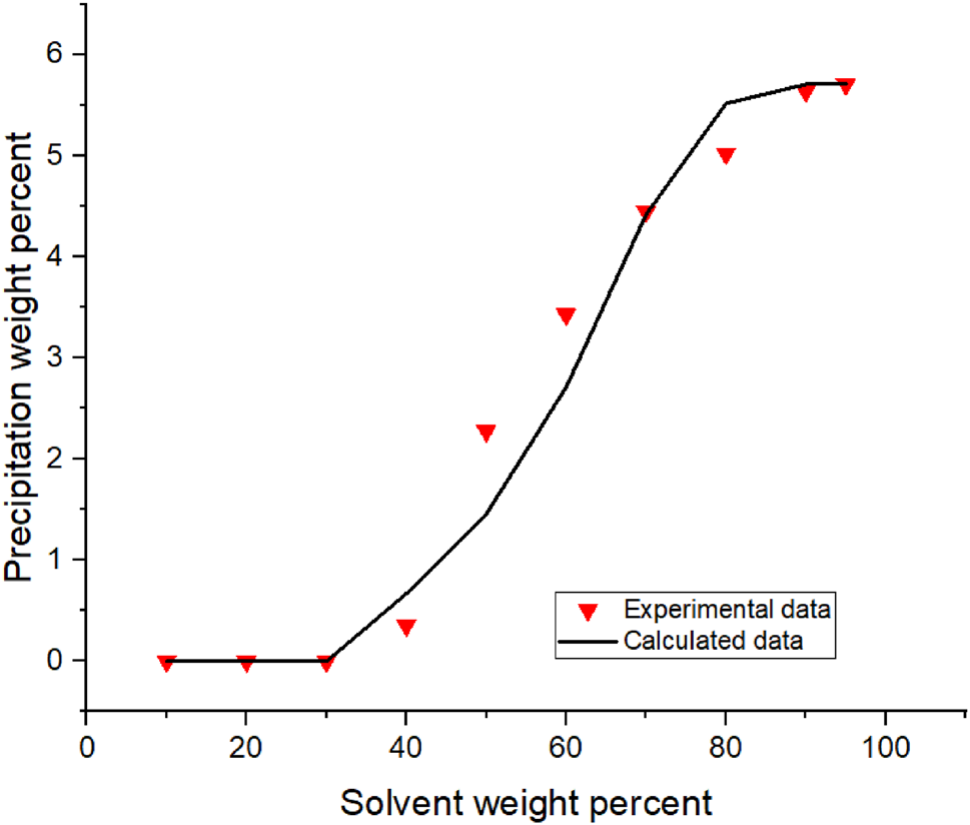
Asphaltene precipitation from the oil sample D versus solvent percent: obtained from experimental and modeling approaches.

**Figure 5:**
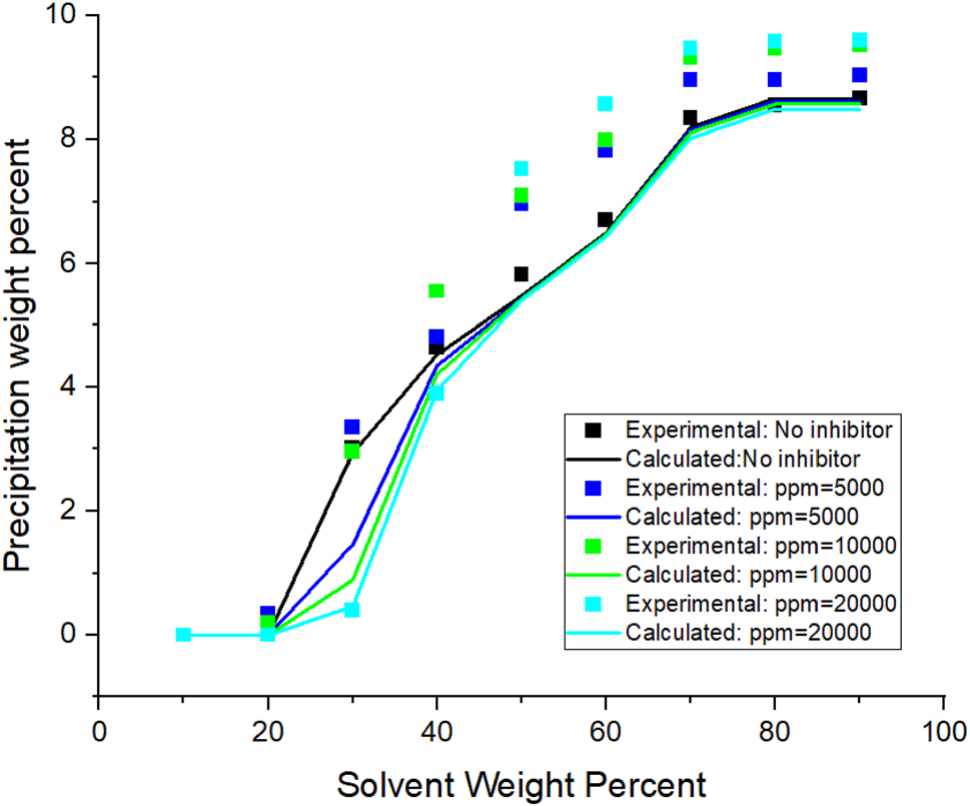
Asphaltene precipitation rate from the oil sample A in terms of solvent ratio with inhibitor (a = 3.38 × 10^11^ and b = 24.51).

**Figure 6:**
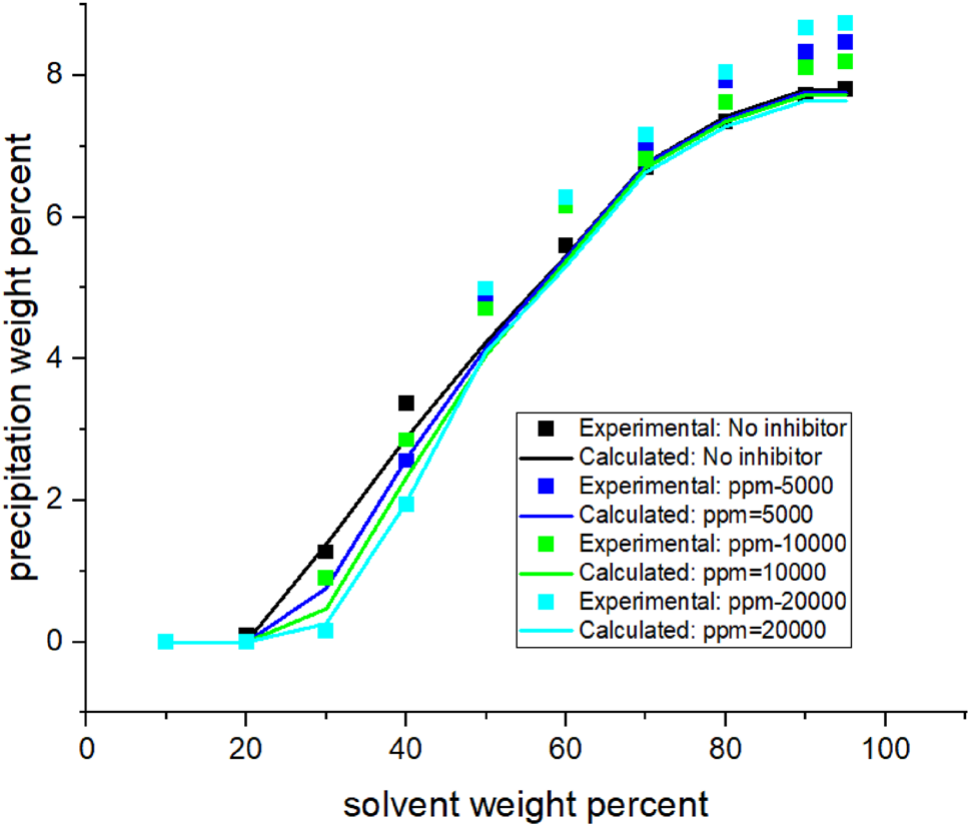
Precipitation rate from the oil sample B in terms of solvent ratio with inhibitor (a = 4.29 × 10^8^ and b = 18.97).

**Figure 7:**
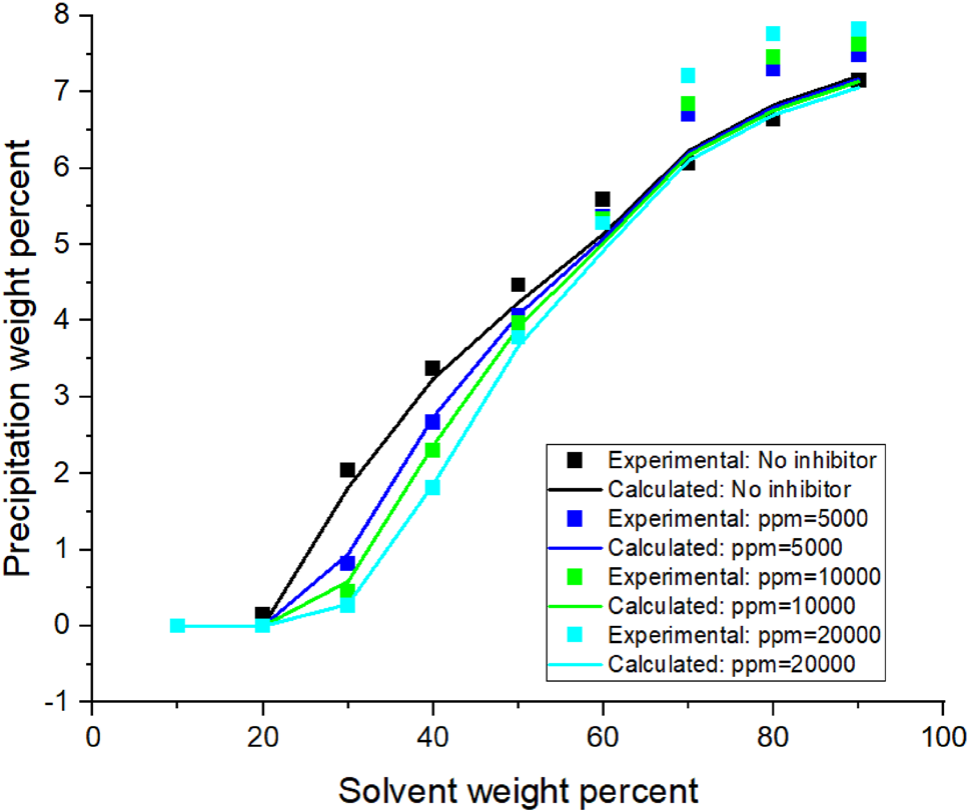
Asphaltene precipitation rate from the oil sample C in terms of solvent ratio with inhibitor (a = 2.17 × 10^6^ and b = 15.06).

**Figure 8:**
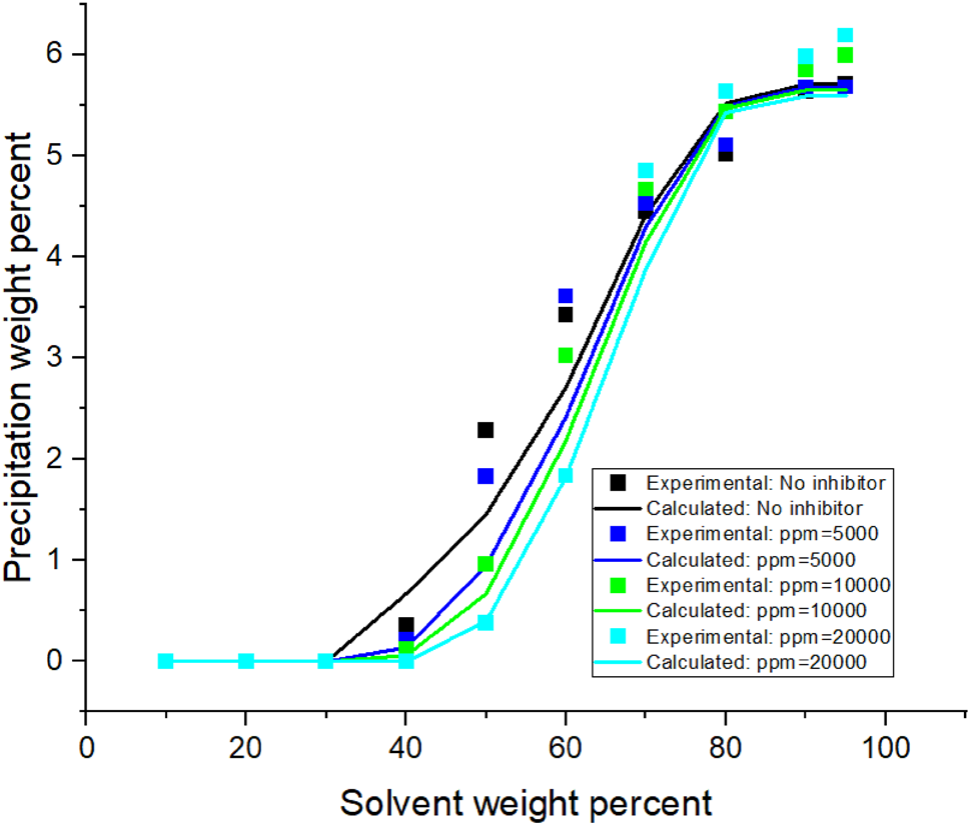
Asphaltene precipitation rate from the oil sample D in terms of solvent ratio with inhibitor (a = 5.28 × 10^7^ and b = 15.69).

### Modeling without inhibitors

The asphaltene precipitation versus solvent ratio is shown in Figs. 1, 2, 3 and 4. According to Figs. 1, 2, 3 and 4, the experimental data are consistent with the values obtained ​​from the modeling approach. As was expected, in all cases, the precipitation rate (the slope of the curves) has its maximum value by adding solvent. This could be due to the oil dilution and the reduction of its solubility parameter. The difference in the solubility parameters of asphaltene and oil increases, leading to the solubility reduction. Decreasing the solubility of two mixed materials enhances phase separation, which is called precipitation. As the solvent ratio increases, more asphaltene molecules precipitate, and as a result, the precipitation rate decreases. The minimum slope of the graph is obtained at high solvent ratios. In addition, the consistency of the experimental data and modeling results is reduced at the endpoints. This occasion might be due to changes in the asphaltene molecular structure, while in the modeling approach, the chemistry of the asphaltene is considered to be unchanged in all the precipitation stages.

As we know, the composition of crude oil samples is different. According to equations two and three, the solubility parameter of the solvent and asphaltene phase depends on the composition of the crude oil. Also, the amount of their asphaltene content is different. As a result, the curves of their asphaltene precipitation are different.

Based on table number 2 in the oil sample D we have less resin content compared to the other oil samples and this causes unstability of asphaltene even though the Asphaltene content of this crude oil sample is lower than the others. As it can be seen from figure number 4 the slope of the curve, or in other words, the rate of precipitation is higher. This fact shows the effect of oil composition and resin content of the sample on the precipitation rate. In compare to this graph, the graphs of oil samples A and C showed a lower rate of precipitation and a higher resin content.

### Modeling in the presence of the inhibitors:

Modeling was conducted in the presence of inhibitors for each oil sample at concentrations of 5000, 10,000, and 2000 ppm. Figures 5, 6, 7 and 8 show the modeling results, in compliance with the experimental results, in the presence of inhibitors at the desired concentrations. As can be seen in Fig. 8, regardless of the type of inhibitor, the precipitation decreases with increasing inhibitor concentration. For instance, asphaltene weight precipitant, at the solvent weight of 50%, is almost 0.3, 0.95, 1.8, and 2.1% for inhibitor concentrations of 20,000, 10,000, 500, and 0 ppm, respectively. The decrease of the precipitation reduces at high solvent ratios. This indicates that increasing the inhibitor and the solvent at the same time will trigger a mechanism that might lead to increasing asphaltene precipitation. Another point in these shapes is the lower graph slope at the beginning of the asphaltene precipitation. This occasion indicates that at lower solvent ratios, the addition of inhibitors leads to delays in precipitation. This fact represents the inhibitor efficiency and its feasibility in reducing asphaltene precipitation. Generally, adding inhibitors to the oil and heptane mixture, as well as increasing the inhibitor concentration, is more effective in lower solvent ratios, but in higher solvent ratios, its effectiveness decreases.

The accommodation between experimental and modelling results is better for crude oil sample D, compared to the samples number A and C. The cause of this occasion and the better working of the model for this oil sample is the difference in composition of the samples. The amount of asphaltene and resin content in sample D , as mentioned before, is less than the other samples. Also adding inhibitors to the oil has better effect on making delay in the onset point for sample D compared to the other ones.

**Figure 9:**
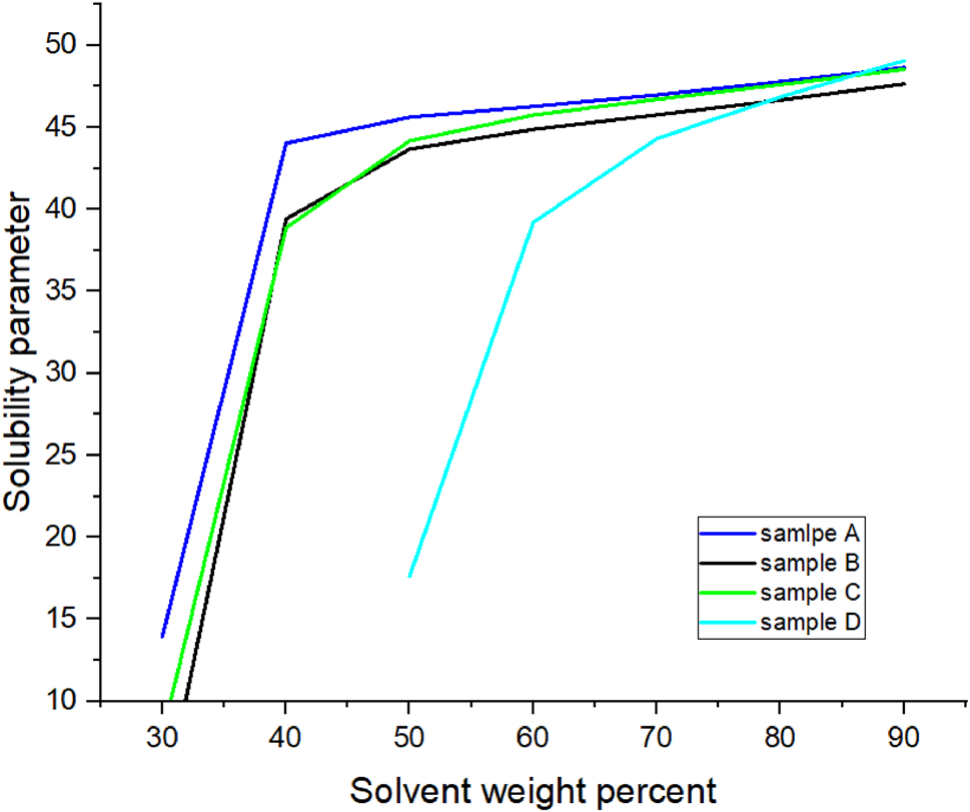
Solubility parameter changes against solvent ratio for different oil samples at 20,000 ppm concentration of inhibitors.

The changes in solubility parameter with adding solvent at 20,000 ppm concentration of inhibitors for several oil samples are plotted in Fig. 9. As it can be seen, by adding solvent, the solubility parameter increases until it reaches about 48 for all kinds of oils.

Based on figure number 9 the solubility parameter of crude oil sample D has lower amount compared to the other samples and as it can be seen from figure number 8 the model on this oil has better accommodation with experimental data. In higher solvent ratios all the compositions for different samples are alike, because of adding lots of amounts of heptane to the samples; so there all of the solubility parameters converge to a certain amount.

### Inhibitor effect on onset point:

Adding an inhibitor changes the values ​​of the Flory–Huggins equation by altering the solubility parameters and shifting the equilibrium condition. This addition could lead to altering the precipitation onset point. As shown in Figs. 5, 6, 7 and 8, the addition of an inhibitor postpones the asphaltene precipitation in all oil samples. In other words, in the presence of an inhibitor, more solvent is required to reach the onset point. This achievement complies with expectations and is consistent with the stabilization of asphaltene in oil.

### Comparison of model and experiment

According to the data obtained from modeling, a comparison between the experimental data and the modeling one was performed. The error calculation is based on the normalized quadratic mean error rate, which is shown in Table 9. As can be seen in Table 7, the highest error, which is 9.8%, is related to oil sample A. The lowest error rate, which is 4.5%, is assigned to oil sample D. It can be seen that the highest amount of asphaltene content is related to oil sample A, and the lowest asphaltene content is assigned to oil sample D. It can be concluded that with increasing the amount of asphaltene in oil, the associated error also increases.

**Table 9:** Calculated errors for different modes of modeling.

Oil sample	Inhibitor concentration (ppm)	Error (%)	Average total error (%)
A	0	2	9.8
	5000	11	
	10,000	13	
	20,000	13	
B	0	3	5.2
	5000	6	
	10,000	5	
	20,000	7	
C	0	3	4.5
	5000	4	
	10,000	5	
	20,000	7	
D	0	7	6.7
	5000	8	
	10,000	6	
	20,000	6	

For each oil sample, the predicted error for cases where the inhibitor is not involved is much lower than the cases where the inhibitor is present. However, with the addition of inhibitors, especially at higher solvent ratios, the error value increases. This occasion could be explained by increasing the precipitation in higher solvent concentrations. As mentioned before, in a higher solvent ratio and the presence of an inhibitor, the asphaltene precipitation increases in a way that is higher than the precipitation without an inhibitor.

## Conclusions

In this study, the efficiency of inhibitors in stabilizing asphaltene, as well as the accuracy of modeling to predict the onset point, ​​was investigated. The most important achievements of this research are as follows:

- Based on the fact that the asphaltene molecule size is larger than other oil molecules, it can be referred to as a polymer. Also, considering the solubility parameter as a key factor showing the precipitation progress, the Flory–Huggins theory that contains this parameter is chosen for modeling asphaltene precipitation.
- In this study, PR EOS was applied, and first, the model is justified in the absence of the inhibitors. Secondly, by adjusting the solubility parameter, precipitation weight percent was predicted.
- The modeling results show that at low solvent ratios, the asphaltene precipitation decreases with the addition of inhibitors.
- According to the modeling results, it can be claimed that the addition of inhibitor at all concentrations postpones the onset point; a higher amount of solvent is required to reach the onset point.
- Increasing the concentration of inhibitors in oil samples enhances its efficiency. For example, the weight of precipitated asphaltene, at a solvent weight of 50%, is almost 0.3, 0.95, 1.8, and 2.1% for inhibitor concentrations of 20,000, 10,000, 500, and 0 ppm, respectively.
- The adjusting parameters in this model for cases where an inhibitor is involved included the volume shift of the components and the Flory–Huggins interaction parameter.
- The proposed model for the cases where an inhibitor is not involved has a better agreement with experimental values.
- Based on the results obtained in this study, it can be concluded that the accuracy of the model is less for situations where the asphaltene content of the crude oil is higher. The average total errors for the oil sample 'A' and 'C' are 9.8 and 4.5%, respectively.

## Supplementary Information

Supplementary Information.

## Data Availability

The datasets used or analyzed during the current study are available from the corresponding author on reasonable request.

## Abbreviations

Mw: Molecular Weight
c: Volume shift
G: Gibbs energy
*H*_*f*_: Fusion enthalpy
W: Weight fraction
x: Molar ratio
P: Pressure
R: Universal gas constant
T: Temperature
U: Internal energy
V: Molar volume
V: Volume
SR: Solvent rate
$$\updelta $$: Solubility parameter
$$\mu $$: Chemical potential
$$\varphi $$: Partial volume
$$\rho $$: Density
A: Asphaltene
S: Solvent
