# Supplementary material for: Modelling the effect of the inhibitors on asphaltene precipitation using Flory–Huggins theory
Source: Sci Rep. 2022 Nov 8;12:18946. doi: 10.1038/s41598-022-23596-w (PMC9643540; doi:10.1038/s41598-022-23596-w)
Supplement: Supplementary file 1 — Supplementary Information. [file 41598_2022_23596_MOESM1_ESM.docx]

**Appendix Figures:**

Calculation of Va from Eq.1 .1

Calculation of δ_a_ from Eq.2

Calculation of new solution composition at each (SR)

Initial guess for volume shift (C1) and calculation of C

Calculation of new MW and Vs using PR

Calculation of δ_a_ using Eq.3

Calculation of Φ_a_ using Eq.4

Calculation of W_cal_ using Eq.5

NO

YES

Print W_cal_

W_cal_ =W_exp_

Figure 1A. The procedure used to predict asphaltene precipitation in the absence of the inhibitors.

**
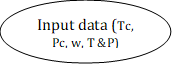
**

Va calculated from PR

a and b

Initial guess from Eq.7

δ from Eq.2

Calculating new molar ratio for new solvent

Calculating Vs & Mws from modified PR

Calculating δ_s_ from Eq.3

Calculating δ_s_ from Eq.8

Calculating Φ_a_ from Eq.7

Calculating W_ca_ from Eq.6

YES

NO

W_cal_ =W_exp_

Print W_cal_

Figure 2A. The procedure used to predict asphaltene precipitation in the presence of the inhibitors

Guessing a0-a3 and calculating I_as_ from Eq. 6

Calculating Φ_a_ from Eq.4

Calculating W_cal_ from Eq.5

YES

NO

Print W_cal_

W_cal_ =W_exp_

Figure 3A. Adjusting Flori-Huggins interaction parameter
